# Individual household-level dynamics and caries experience among young children in peru. a cross-sectional study

**DOI:** 10.21142/2523-2754-1302-2025-240

**Published:** 2025-05-16

**Authors:** Luis Limo, Lupe Antonieta Vargas-Zafra, Ronald Espíritu Ayala-Mendívil

**Affiliations:** 1 Schulich School of Medicine and Dentistry, Western University. London, Ontario, Canada. llimo@uwo.ca Schulich School of Medicine and Dentistry Western University London, Ontario Canada llimo@uwo.ca; 2 Preventive Medicine and Public Health, Universidad Nacional Mayor de San Marcos. Lima, Peru. lvargasz@unmsm.edu.pe, rayalam@unmsm.edu.pe Universidad Nacional Mayor de San Marcos Preventive Medicine and Public Health Universidad Nacional Mayor de San Marcos Lima Peru lvargasz@unmsm.edu.pe rayalam@unmsm.edu.pe

**Keywords:** family, oral health, preschool, home environment, salud bucal, relaciones familiares, salud infantil, estructura familiar

## Abstract

**Objective:**

We aimed to assess the association between household dynamic factors and early childhood caries (ECC) experience, and whether these associations differ based on the extent of dental tissue damage.

**Materials and methods:**

We analyzed data from 210 children aged 3-5 years in Callao, Peru, using questionnaires and dental examinations. Household dynamics included house ownership, parental living arrangements, family structure, and family size. ECC experience was assessed by clinically examining decayed, filled, or missing primary teeth, including cavitated and non-cavitated lesions. Covariates included demographic, socio-economic, health behaviours, and access to dental care. Block-wise multivariable regression models with prevalence ratios and 95% CI were used, following STROBE guidelines.

**Results:**

Fully adjusted estimates showed that the prevalence of ECC experience was higher among children living in rented accommodations (PR 1.29, 95% CI 1.17, 1.41), in single-parent households (PR 1.75, 95% CI 1.61, 1.91), with extended family beside their nuclear family (PR 1.19, 95% CI 1.08, 1.29), and sharing the household with at least 4 other members (PR 1.05, 95% CI 0.94, 1.14). Sub-group analysis suggested that family structure and family size were significantly associated with non-cavitated dental lesions, while the association with cavitated dental lesions was stronger when associated with parental living arrangements, after controlling for all covariates.

**Conclusions:**

Individual household-level dynamics indicators are associated with the early onset of dental caries among young children in Peru. However, further analysis is required to fully understand the underlying mechanisms and other contributing factors of ECC in this study population.

## RESUMEN

**Objetivos:**

El objetivo fue evaluar la asociación entre las dinámicas individuales del entorno familiar y la experiencia de caries de aparición temprana (CAT), así como determinar si estas asociaciones varían según el grado de afectación del tejido dental.

**Materiales y métodos:**

Se analizaron datos de 210 niños de 3 a 5 años en Callao, Perú, a partir de cuestionarios y exámenes dentales. Las dinámicas familiares evaluadas incluyeron la propiedad de la vivienda, la convivencia parental, la estructura familiar y el tamaño de la familia. La experiencia de CAT se evaluó mediante el examen clínico de los dientes primarios cariados, obturados o ausentes, diferenciando las lesiones cavitadas y no cavitadas. Las covariables fueron demográficas, socioeconómicas, conductas de salud y acceso a los servicios odontológicos. Para el análisis, utilizamos modelos de regresión multivariable por bloques, estimando cocientes de prevalencia e intervalos de confianza al 95%, y reportamos nuestros hallazgos de acuerdo con las directrices STROBE.

**Resultados:**

Los modelos totalmente ajustados indicaron que la prevalencia de experiencia de CAT fue mayor en niños que residían en viviendas alquiladas (RP 1,29; IC 95% 1,17; 1,41), en hogares monoparentales (RP 1,75; IC 95% 1,61; 1,91), en familias extensas además de la familia nuclear (RP 1,19; IC 95% 1,08; 1,29) y en aquellos que compartían el hogar con al menos cuatro miembros adicionales (RP 1,05; IC 95% 0,94; 1,14). El análisis de subgrupos de acuerdo con el grado de afectación del tejido dental mostró que la estructura familiar y el tamaño del hogar estaban significativamente asociados con lesiones no cavitadas, mientras que la asociación con lesiones cavitadas fue más pronunciada en relación con la convivencia parental, después de ajustar por todas las covariables.

**Conclusiones:**

Los indicadores relacionados con las dinámicas individuales del entorno familiar están asociados con la experiencia de caries de aparición temprana (CAT) en niños pequeños en el Perú. Sin embargo, se requieren análisis adicionales para comprender plenamente los mecanismos subyacentes y otros determinantes que contribuyen al desarrollo de la caries en esta población de estudio.

## INTRODUCTION

Oral diseases are a major public health concern, as the recent WHO Global Oral Health Status estimated that they affect close to 3.5 billion people worldwide, with dental caries being the most prevalent chronic diseases in children, particularly affecting those coming from lower socio-economic positions ^1^. Importantly, the consequences of early childhood caries (ECC), defined as the presence of a primary tooth with one or more caries lesions (cavitated or non-cavitated), missing (due to caries), or filled surfaces in a child under the age of six years ^2^, include negative impacts on growth, development, nutritional problems, and oral health-related quality of life issues ^3^. Previous studies have suggested that children with dental caries tend to have poorer school performance and lower school attendance rates ^4^. Particularly, last available data from Peru, shows that high rates of ECC have been reported, revealing significant oral health inequalities, with a 59.1% of children with primary dentition diagnosed with dental caries ^5^. 

Similar to other chronic conditions, dental caries is recognized as a multifactorial disease, influenced by an interplay of biological, health behaviours, and socio-environmental factors ^6^^,^^7^. Among these, household dynamics play a crucial role in shaping children's oral health outcomes, particularly in vulnerable populations ^8^. Household dynamics, including parental living arrangements, family structure, household ownership, and family size, are hypothesized to be determinants of a child's oral health ^9^^-^^11^. According to the 2017 Demographic and Familiar Health Survey (ENDES), in Peru, the family dynamic and household structure vary largely based on social classes and geographic regions. For instance, the nuclear family is the most common household unit and varies in size depending on the family’s geographic location. A significant proportion of children live with both parents, although there is a growing trend of single-parent households, particularly in urban areas ^12^. Lima households often consist of nuclear families, but there is a growing number of single-parent and extended family households, with an average family size of approximately 3.5 members. Around 30% of households have children under the age of 18, and single-parent households, primarily headed by women, make up about 20% of all households with children ^12^.

The Social Determinants of Health (SDH) framework, posits that health outcomes are influenced by the conditions in which people are born, grow, live, work, and age ^13^. According to this framework, socio-environmental factors including family interactions are pivotal in determining health outcomes, including oral health ^14^. Several frameworks addressing childhood health development have integrated family functioning as a crucial element theoretically impacting the development of children's oral health as well as the adoption of preventive oral health behaviours ^15^^,^^16^. The family’s role has predominantly been examined from behavioural, cognitive, and to a lesser extent, psychosocial perspectives, focusing on aspects such as parental oral health practices, knowledge, attitudes, and social status, rather than specific household dynamic interactions. Previous research suggest that close family interactions, characterized by supportive and cohesive relationships, may be associated with healthy behaviours, and subsequently, provide emotional and practical support for maintaining oral hygiene practices ^17^^,^^18^. However, little is known whether household dynamics, as the primary and fundamental socio-environmental factor in an individual’s life, could have an impact on the early onset of dental caries in young children. The aim of our study was to estimate the extent of the association between each of the individual-household level dynamics indicators and ECC experience among children aged 3-5 years old living in the province of Callao, Peru, and whether these factors are associated with the dental tissue structure’s damage. We hypothesize that there is a relationship between each of these factors and the caries experience among young children. 

## MATERIALS AND METHODS

The study followed ethical guidelines, including the 1964 Declaration of Helsinki and its updates. It was approved by the Graduate Studies Committee at San Fernando - Faculty of Medicine, Universidad Nacional Mayor de San Marcos (No. 018-UPG-FM-02018) and authorized by both the Regional Directorate of Education of Callao (DREC, Peru) and the school administration. Parents or legal guardians of eligible children provided written informed consent before participation. Data collection was anonymous, and parents completed questionnaires about their household and socio-demographic details. Our study follows STROBE reporting guidelines (see Supplementary Material).

### Measurement of Variables

Household dynamics: We used 4 indicators including house ownership, parental living arrangements, family structure, and family size. House ownership was defined according to whether the family owns or rents the home in which the child resides. Parental living arrangements were dichotomized as whether the caregiver reports as a single parent (mother, or father), or whether the child lives with both of their parents. The variable for family structure distinguishes between nuclear families and extended families. A nuclear family consists of at least one parent and their children living together as a single household unit. In contrast, an extended family includes not only the nuclear family members but also other relatives such as grandparents, aunts, uncles, and cousins living in the same household and maintaining close daily interactions. Considering the latest census conducted in Peru, showing that the average family size in Lima is approximately of 3.5 members, we defined the variable family size as ‘small’ for households with up to 3 members, and as ‘large’ for those with 4 or more members.

Caries experience: This procedure was performed by a registered dentist, using portable equipment, dental explorers, biosafety measures, with support from a dental assistant as a note-taker, in a suitable room provided by the school, and it took approximately 10 minutes per child. We defined caries experience as the presence of at least one caries lesion (cavitated or non-cavitated), or filled surfaces, or missing teeth due to dental caries. Then, we dichotomized this variable into ‘high’ and ‘low’ using the 75^th^ percentile as a cut-off point, given the high prevalence (>10%). Specifically, we categorized children with a dmft score above the 75^th^ percentile as having high caries experience, and those with a dmft score below or equal to the 75^th^ percentile as low caries experience. For our sub-group analysis, dental lesions manifested as distinct, chalky white enamel, or with no clinically visible lesions, such as white spot lesions, were defined as non-cavitated. In contrast, cavitated lesions corresponded to those with evident loss of enamel structure ^19^. Our criteria did not differentiate between cavitated enamel and dentinal lesions. 

**Figure 1:**
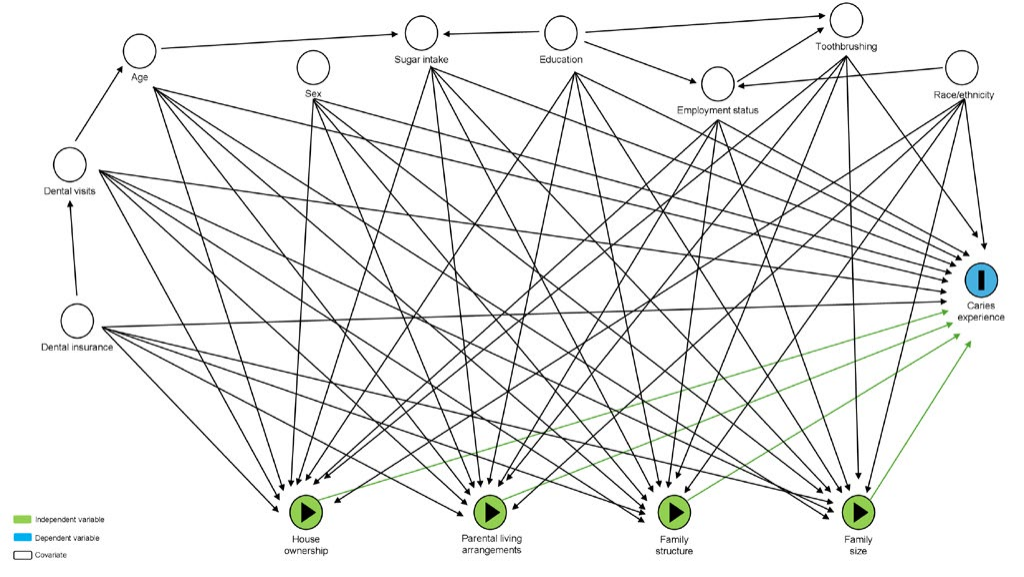
The directed acyclic graph (DAG) shows the potential association between several household dynamic indicators, such as house ownership, parental living arrangements, family structure, and family size, as independent variables, and caries experience as outcome of interest. Multiple covariates such as demographic, socio-economic, health behaviours, and factors of access to dental services may confound these associations.

Covariates: These included demographic factors such as child’s age, sex, and race/ethnicity. Parent’s socio-economic characteristics included their level of education, and employment status. Health behaviour factors included assisted toothbrushing, and frequency of sugar intake which was defined as high when 6 or more teaspoons of added sugar consumed daily, following the recommendation by the American Academy of Pediatrics (AAP) stating that children aged 2 years and older should consume less than 25 grams (approximately 6 teaspoons) of added sugar per day ^20^. Other covariates included factors related to access to dental services. These factors encompass the type of dental insurance, which is dichotomized as either public or private, and whether the child has ever visited a dentist for any reason. (Figure 1) 

### Statistical analysis

We employed descriptive statistics reporting in absolute numbers and percentages for all variables. Next, multivariable block-wise regression models were constructed to estimate prevalence ratios (PR) with 95% confidence intervals (CI) of the association between household dynamics indicators and ECC experience, controlling for the aforementioned covariates. For the sub-group analysis, we summed the total number of decayed teeth, and stratified them by cavitated, and non-cavitated lesions as outcomes of interest, and adjusting for all covariates. All analyses were conducted using Stata v18.0 statistical software ^21^. 

## RESULTS

### Characteristics of the study population

The sample comprised 210 children, each corresponding to one household, almost equally distributed by their age, with most being 3 years old (35.7%), followed by those aged 5 and 4 years. Most of the children were female (57.1%) and identified as mixed (78.6%). Predominantly, they were raised by single parents (77.1%), shared the household with at least 4 members (58.1%), and lived in rented accommodations (81.9%). Most of the households were defined as extended families (69.1%), including grandparents, aunts, uncles, and other relatives. Notably, 31.4% of the parents reported having incomplete high school education, and most (90.5%) were currently working. About 73.1% of the study sample was diagnosed with a decayed, missing, or filled tooth due to caries. Among those with decayed lesions, most had cavitated lesions (59.2%). While most of the parents reported not assisting their children during toothbrushing (89%) and never taking their child to the dentist (87.6%), 172 out of the 210 examined children fell into the category of low ECC caries experience (81.9%) compared to those with high ECC caries experience (18%). (Table 1).

**Table 1:** Descriptive statistics of characteristics of study participants (n = 210) stratified, Callao, Peru, 2017.

Variables			All n (%) 210 (100)
Age	3y		75 (35.7)
	4y		67 (31.9)
	5y		68 (32.4)
Sex	Males		90 (42.9)
	Females		120 (57.1)
Race / Ethnicity	White		35 (16.7)
	Mixed		165 (78.6)
	Black		10 (4.7)
DMFT index	Decayed	Cavitated	64 (30.4)
		Non-cavitated	44 (20.9)
	Missing		36 (17.1)
	Filled		10 (4.7)
	No caries		56 (26.6)
Early childhood caries experience	High (> 75th percentile)		38 (18)
	Low (≤ 75th percentile)		172 (81.9)
Household dynamics	House ownership	Own	38 (18.1)
		Rented	172 (81.9)
	Parental living arrangements	Single parents (single mother, or single father)	162 (77.1)
		Two-parent household	48 (22.9)
	Family structure	Nuclear family	65 (30.9)
		Extended family	145 (69.1)
	Family size	Small (up to 3 members)	88 (41.9)
		Large (4 or more members)	122 (58.1)
Health behaviours	Toothbrushing	Assisted	23 (11)
		Not assisted	187 (89)
	Sugar intakea	High	91 (43.3)
		Low	119 (56.7)
Parents’ socio-economic characteristics	Education level	Incomplete high school	66 (31.4)
		Complete high school or post-secondary education	144 (68.6)
	Employment status	Active	190 (90.5)
		Inactive	20 (9.5)
Access to dental services	Type of health insurance	Public (including army forces-funded)b	190 (90.4)
		Private	20 (9.6)
	Ever visited the dentist	Yes	26 (12.4)
		No	184 (87.6)

a High sugar intake: ≥ 6 teaspoons of sugar daily; ^b^Government-funded including Essalud and the National Health Insurance (SIS by its acrnomym in Spanish)

### Household dynamics indicators associate with high ECC experience

Each individual-household level dynamics indicator was significantly associated with young children’s caries experience. Parental living arrangement was the strongest indicator associated with a high ECC experience, followed by families in which the child, in addition to living with at least one parent, also lived with other relatives such as aunts, uncles, grandparents, cousins, etc. For example, the prevalence of having high ECC experience was almost twice as high in those children living with only one of their parents (PR 1.75, 95% CI 1.61, 1.91), after adjusting for other covariates. Specifically, this association was mostly explained by socio-economic factors such as the caregiver’s education level and employment status, contributing to 52% of the association. Other indicators, such as living in a rented house (PR 1.78, 95% CI 1.61, 1.87), living with other family members (PR 1.93, 95% CI 1.80, 2.12), and sharing the household with at least 4 other members (PR 1.39, 95% CI 1.26, 1.50) were significantly associated with high ECC experience. (Table 2).

**Table 2:** The association between household level dynamics indicators and high early childhood caries (ECC) experience, Callao, Peru, 2017.

	PR0 (95% CI)	PR1 (95% CI)	PR2 (95% CI)	PR3 (95% CI)	PR4 (95% CI)
House Ownership (Rented)	1.78* (1.61, 1.87 )	1.75* (1.62, 1.89)	1.63* (1.49, 1.79)	1.32* (1.21, 1.47)	1.29* (1.17, 1.41)
Parental Living Arrangements (Single Parents)	2.79* (2.59, 2.93)	2.71* (2.54, 2.88)	2.19* (2, 2.27)	1.89* (1.71, 1.96)	1.75* (1.61, 1.91)
Family Structure (Extended Family)	1.93* (1.80, 2.12)	1.90* (1.79, 2.07)	1.77* (1.61, 1.85)	1.32* (1.23, 1.41)	1.19* (1.08, 1.29)
Family Size (Large)	1.39* (1.26, 1.50)	1.40* (1.26, 1.49)	1.30* (1.22, 1.41)	1.15* (1.07, 1.22)	1.05 (0.94, 1.14)

*p values ≤0.05, PR: prevalence ratio, CI: confidence interval

Model 0: Crude estimates (unadjusted)

Model 1: Adjusting for age, sex, race/ethnicity

Model 2: In addition, adjusted for parents’ education level, and employment status

Model 3: In addition, adjusted for assisted toothbrushing, and sugar intake

Model 4: In addition, adjusted for type of dental insurance, and ever visited the dentist

### Sub-group analysis stratified by cavitated and non-cavitated dental lesions

**Figure 2:**
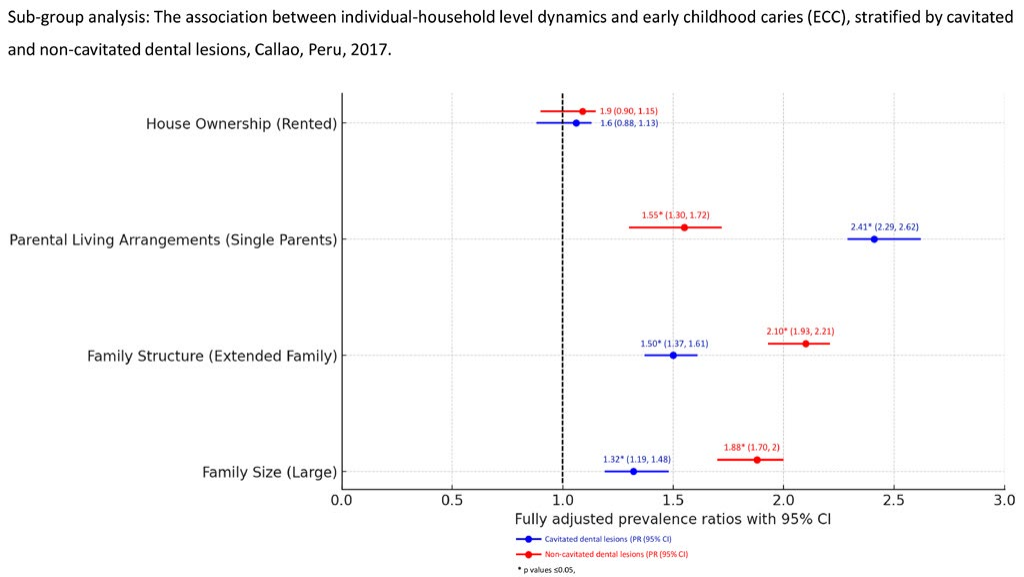
Forest plot graph shows the extent of the associations between each of the household dynamic indicators and cavitated (blue lines) and non-cavitated dental lesions (red lines), reported in prevalence ratios (PR) using 95% confidence intervals (CI). In our study sample, we found significant associations for each of the household dynamic indicators, except for house ownership, indicating that living in rental accommodations is not associated with the prevalence of either cavitated or non-cavitated dental lesions.

Sub-group analysis revealed that several household dynamics indicators such as parental living arrangements, family structure, and family size were significantly associated with dental caries progression. The fully adjusted estimates controlling for demographic, socio-economic, health behaviours, and factors of access to dental services, suggest that the prevalence of having non-cavitated dental caries, such as white spot lesions, was higher among those children living in an extended family structure or sharing the household with at least 4 other members (PR 2.10, 95% CI 1.93, 2.21, and PR 1.88, 95% CI 1.70, 2, respectively), compared to their counterparts. Conversely, children living in single-parent families had a 2.4 times higher prevalence of having cavitated dental lesions compared to those with non-cavitated ones (PR 2.41, 95% CI 2.29, 2.62). No differences were noted when the associations were with house ownership. (Figure 2).

## DISCUSSION

By cross-sectionally analyzing data from 210 children representing their households, we found that house ownership, parental living arrangements, family structure, and family size were significantly associated with a higher prevalence of ECC experience. Estimates from further analysis revealed that the prevalence of cavitated dental lesions was higher among young children from single-parent families, living with more members beyond their nuclear family, and sharing the household with at least four members. Children from larger families tended to have a higher prevalence of caries experience, in part due to resource dilution, where the attention and resources available per child decrease as the number of children increases. The rationale behind the association between the number of family members and caries experience may be explained through several mechanisms. Larger households may face greater financial constraints, leading to limited access to dental services and oral hygiene products. Additionally, in larger families, parents might have less time to dedicate to each child's oral hygiene, resulting in inadequate toothbrushing and oral hygiene routines ^22^. Moreover, siblings may share eating habits and sugary snacks, contributing to a higher risk of dental caries ^23^^,^^24^. Furthermore, a study conducted in families with 3- and 4-year-old children found that parents' marital status, including single-parent households, correlates with increased caries incidence, likely due to reduced supervision and inconsistent oral hygiene practices ^9^. Our estimates indicate that children raised by single parents showed a higher prevalence of caries experience compared to those raised by both parents. Single-parent households, due to economic struggles, increased stress, and limited time for childcare, may face challenges in maintaining regular dental visits and consistent oral hygiene routines for their children, potentially impacting overall health behaviours ^23^^,^^25^. Estimates from our sub-group analysis on the extent of dental tissue damage may be partly explained by the notion that these households may have fewer resources for preventive measures, such as regular dental visits. Consequently, the lack of timely intervention and ongoing dental care can lead to the progression of non-cavitated lesions, such as white spot lesions, into cavitated lesions, indicating more advanced tissue destruction ^26^^,^^27^.

Our findings strengthen our hypothesis that children living with their nuclear families may be generally less likely to develop dental caries compared to those sharing households with extended relatives. This difference may stem from more consistent caregiving routines in nuclear families, where parents can closely monitor and enforce oral hygiene practices. Extended family members might introduce varied dietary habits and potentially less rigorous oral hygiene standards, increasing caries risk among children ^28^. 

Our sub-group analysis indicates that children living with extended family and in households with at least four members have a higher prevalence of early dental caries, such as non-cavitated lesions. This may be due to shared resources and differences in dietary and hygiene practices. Extended families often expose children to more sugary foods and beverages, while larger families might experience resource dilution, reducing attention and financial support for each child's dental care ^6^^,^^29^. However, we recognize that other variables related to breastfeeding, oral health habits during pregnancy, and use and frequency of bottle-feeding could potentially influence ECC experience ^30^^,^^31^. 

One of the strengths of this study was the use of four comprehensive indicators of household dynamics (house ownership, parental living arrangements, family structure, and family size), providing a thorough understanding of the family environment. Second, the use of reliable and valid instruments to measure both family dynamics factors and caries experience, including the validation of the questionnaire using the Cronbach alpha test, adds to the study's robustness. Third, the use of clinical examinations for ECC provides an accurate assessment of children’s oral health. The clinical assessment conducted by a registered dentist ensures reliability and accuracy in diagnosing caries. Finally, the identification of multiple covariates acting as potential confounders and controlling them by the use of multivariable block-wise regression models for statistical analysis provides more precise estimates of the associations between household dynamics and ECC experience.

We acknowledge several limitations. First, the cross-sectional design limits the ability to draw causal inferences, as it captures data at a single point in time without considering changes over time. Second, the sample size, while sufficient for detecting associations, may not be large enough to generalize the findings to broader communities in Peru or other similar settings. Third, the reliance on questionnaires for data collection may introduce the risk of reporting bias, as responses may be influenced by social desirability or recall bias. Fourth, the clinical assessment, although thorough, did not differentiate cavitated enamel lesions from those involving dentin, potentially limiting the granularity of the caries experience data. However, while the dmft index does not allow us to assess the severity of the dental caries lesion, our clinical assessment allowed us to record the type of lesion by differentiating between cavitated and non-cavitated lesions, indicating the degree of progression of dental caries, particularly relevant in primary dentition. Fifth, our methods of dichotomizing certain variables, such as family size and caries experience, may oversimplify the complexity of these factors and reduce the variability in the data. Furthermore, the potential for unmeasured confounding variables, despite the inclusion of numerous covariates, remains a concern, as other factors influencing caries experience may not have been accounted for. Finally, the study's findings, while aligning with previous research, may still be subject to contextual biases specific to the studied population and setting, necessitating caution when generalizing to broader populations.

Our study has several policy implications, including the development of targeted interventions that address the specific needs of high-risk groups, such as single-parent households and larger families, by providing additional resources and support to mitigate the economic and social challenges these families face. Particularly in Peru, enhancing access to dental services and promoting educational campaigns by integrating oral health education into broader family support programs could be beneficial. Future research should focus on longitudinal studies to establish causal relationships and better understand the long-term effects of household dynamics on children’s oral health. Additionally, exploring the impact of other variables, such as breastfeeding practices, use of bottle-feeding, and maternal oral health habits during pregnancy, could provide a more comprehensive understanding of the factors influencing ECC. Ultimately, expanding the study to include diverse populations and settings will also help to generalize the findings and inform more effective public health strategies globally.

## CONCLUSION

Individual household-level dynamics factors, including house ownership, parental living arrangements, family structure, and family size, are associated with the early onset of dental caries among young children in Peru. While parental education level and employment status explain most of these associations, further analysis is required to fully understand the underlying mechanisms and other contributing factors. 
